# Fertility Desires and Intentions of HIV-Positive Women of Reproductive Age in Ontario, Canada: A Cross-Sectional Study

**DOI:** 10.1371/journal.pone.0007925

**Published:** 2009-12-07

**Authors:** Mona R. Loutfy, Trevor A. Hart, Saira S. Mohammed, DeSheng Su, Edward D. Ralph, Sharon L. Walmsley, Lena C. Soje, Marvelous Muchenje, Anita R. Rachlis, Fiona M. Smaill, Jonathan B. Angel, Janet M. Raboud, Michael S. Silverman, Wangari E. Tharao, Kevin Gough, Mark H. Yudin, for the Ontario HIV Fertility Research Team

**Affiliations:** 1 Women's College Research Institute, Women's College Hospital, Toronto, Ontario, Canada; 2 Department of Medicine, University of Toronto, Toronto, Ontario, Canada; 3 Department of Psychology, Ryerson University, Toronto, Ontario, Canada; 4 Dalla Lana School of Public Health, University of Toronto, Ontario, Canada; 5 University Health Network, Toronto, Ontario, Canada; 6 Infectious Diseases Care Program, St. Joseph's Health Care, London, Ontario, Canada; 7 Black Coalition for AIDS Prevention, Toronto, Ontario, Canada; 8 Women's Health in Women's Hands, Toronto, Ontario, Canada; 9 Sunnybrook Health Sciences Centre, Toronto Ontario, Canada; 10 McMaster University Medical Centre, Hamilton Health Sciences, Hamilton, Ontario, Canada; 11 Ottawa Health Research Institute and The Ottawa Hospital, Ottawa, Ontario, Canada,; 12 Lakeridge Health Centre, Oshawa, Ontario, Canada; 13 Department of Medicine, St. Michael's Hospital, University of Toronto, Toronto, Ontario, Canada; 14 Department of Obstetrics and Gynecology, St. Michael's Hospital, University of Toronto, Toronto, Ontario, Canada; University of Cape Town, South Africa

## Abstract

**Background:**

Improvements in life expectancy and quality of life for HIV-positive women coupled with reduced vertical transmission will likely lead numerous HIV-positive women to consider becoming pregnant. In order to clarify the demand, and aid with appropriate health services planning for this population, our study aims to assess the fertility desires and intentions of HIV-positive women of reproductive age living in Ontario, Canada.

**Methodology/Principal Findings:**

A cross-sectional study with recruitment stratified to match the geographic distribution of HIV-positive women of reproductive age (18–52) living in Ontario was carried out. Women were recruited from 38 sites between October 2007 and April 2009 and invited to complete a 189-item self-administered survey entitled “The HIV Pregnancy Planning Questionnaire” designed to assess fertility desires, intentions and actions. Logistic regression models were fit to calculate unadjusted and adjusted odds ratios of significant predictors of fertility intentions. The median age of the 490 participating HIV-positive women was 38 (IQR, 32–43) and 61%, 52%, 47% and 74% were born outside of Canada, living in Toronto, of African ethnicity and currently on antiretroviral therapy, respectively. Of total respondents, 69% (95% CI, 64%–73%) desired to give birth and 57% (95% CI, 53%–62%) intended to give birth in the future. In the multivariable model, the significant predictors of fertility intentions were: younger age (age<40) (p<0.0001), African ethnicity (p<0.0001), living in Toronto (p = 0.002), and a lower number of lifetime births (p = 0.02).

**Conclusions/Significance:**

The proportions of HIV-positive women of reproductive age living in Ontario desiring and intending pregnancy were higher than reported in earlier North American studies. Proportions were more similar to those reported from African populations. Healthcare providers and policy makers need to consider increasing services and support for pregnancy planning for HIV-positive women. This may be particularly significant in jurisdictions with high levels of African immigration.

## Introduction

Indisputable health improvements have occurred with the advent of antiretroviral therapy (ART) over the last 15 years, resulting in dramatic reductions in HIV-related morbidity and mortality and yielded improvements in quality of life [1]. Breakthroughs in pregnancy care have reduced the risk of vertical transmission to <1% if pregnant women receive timely ART, achieve optimal viral suppression, deliver by caesarean-section when appropriate, and avoid breastfeeding [2]. Over the past several years, the worldwide HIV epidemic has reached gender parity, and in parallel, the Canadian demographics have shifted towards increased rates of females being infected [3], [4]. Twenty-eight percent of all new HIV infections in Canada are diagnosed in women, who now represent 17% of Canada's HIV-positive population [4]. Furthermore, over 80% of HIV-positive women are of reproductive age [4].

This combination of factors suggests that many HIV-positive women may desire to become pregnant and that pregnancy planning will become an increasingly important component of HIV medicine. It is important to understand the fertility desires and intentions of the current generation of HIV-positive women in order to develop programs to support them and their current and future counterparts in planning safer pregnancies that protect the health of the women, their partners and their children.

Previous studies addressing the fertility intentions of HIV-positive women have been important but some are of limited scope [5]–[33]. Most were conducted before the widespread use of combination ART, or included only small samples of women [20]–[33]. Others examined fertility choices in selected populations such as drug using, Latina, Asian, South Asian, Caucasian, and Aboriginal women [7]–[9], [11], [32]. Consequently, we developed this study to engage a larger number of women living in Ontario, Canada and to sample a population that uniquely reflects the global community of women living with HIV. Due to Ontario's openness to immigration and the large community of African refugees, population-based observational HIV studies carried out in Ontario can provide important insights relevant to similar centres worldwide.

This cross-sectional study was designed to gather information about the fertility desires, intentions and actions of HIV-positive women, and to identify predictors of fertility intentions. By collecting information from women of reproductive age in communities across Ontario, we hope to aid healthcare providers and policy makers in identifying gaps and in planning for needed services.

## Methods

### Ethics Statement

The overall study received ethics approval from the Women's College Research Institute Research Ethics Board and each research site received ethics approval from their local institutional research ethics board prior to commencement of the study at their site. Written informed consent was obtained from every participant prior to the start of any research activities.

### Study Design and Population

**Figure 1:**
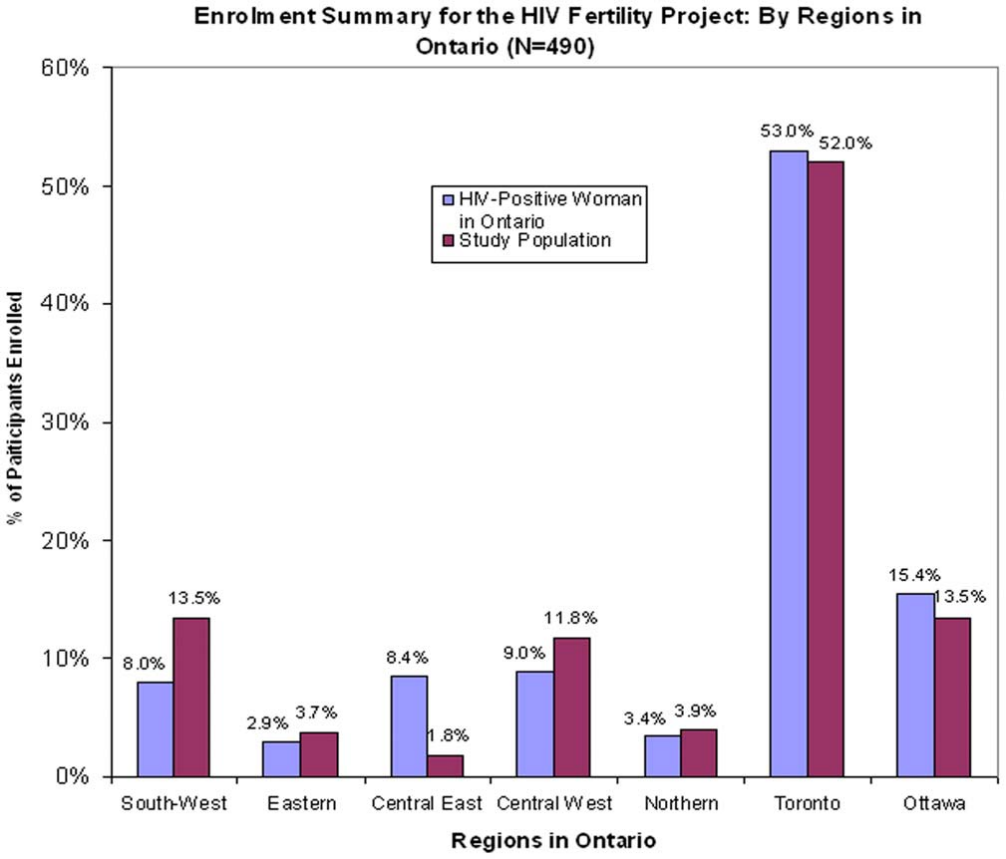
Comparison of regional distribution of study population to the regional distribution of Ontario HIV-positive female population from the 2006 epidemiologic data by provincial region laid out by the provincial Public Health [35]. Figure footnote: While target recruitment was 525, only 493 were recruited and only 490 met the inclusion criteria for analysis. Recruitment was halted due to futility of recruitment in one provincial region with low recruitment. The chi-square test p-values comparing the distribution of the sample and the total HIV-positive female population in Ontario according to geographic unit was <0.05 due to two over-enrolling regions and one under-enrolling region. However, the main reason for the regional representation of enrolment was to ensure that not all participants were from Toronto. When the chi-square test is carried out to assess Toronto vs. non-Toronto participants, the p-value is 0.6.

A cross-sectional study using a survey instrument was carried out with participants who met the following inclusion criteria: 1) HIV-positive, 2) biologically female, 3) of reproductive age (between the ages of 18 and 52), 4) living in Ontario, Canada, and 5) ability to read English or French. The upper age limit was chosen to reflect the cut-off for fertility clinic consultation in Canada. A planned sample size of 525 was calculated in order to estimate the proportion of women intending to have children in the future with a 95% confidence interval (CI) of ≤ ±5%. Recruitment was conducted from October 5^th^, 2007 to March 31^st^, 2009 through 28 AIDS service organizations (ASOs), eight primary care and specialty HIV clinics, and two community health centres (CHCs) across the province of Ontario. An invitation email was sent out to all Ontario ASOs listed on the Canadian AIDS Society website (the national umbrella ASO) and all clinics and CHCs known by the investigators that care for HIV-positive women (56 invited sites, 12 declined, 6 agreed and did not recruit). Recruitment and study qualification determination was carried out by a single assigned research staff at each ASO, clinic and CHC following a pre-developed recruitment plan (available upon request) [34]. The recruitment was carried out in a consecutive manner as research staff were instructed to invite every consecutive qualifying woman who received services in their centre or clinic on all days that care was provided to participate. While records were not maintained on the exact numbers of candidates who were eligible, approached and agreed to participate, coordinators were asked to record the numbers that declined and the reason. An attempt was made for the recruitment to be stratified such that the study sample would be proportional to the geographic distribution of HIV-positive female population in Ontario [35]. The geographic unit was identified as the provincial regions laid out by the provincial Public Health and the specific regions are noted in Figure 1 and Table 1. This non-random sampling technique was used in an effort to obtain a representative sample of HIV-positive women of reproductive age living in Ontario in the absence of a registry of HIV-positive which, if available, would have allowed for a random sample. Bi-annual advisory meetings were held with investigators, coordinators and community representatives in order to monitor and discuss the actual and targeted recruitment for each region and subsequent regional recruitment strategies were accordingly implemented. Once written consent was obtained, the survey could be completed at the site or at home. If a participant responded that she had previously filled out the questionnaire at another site, her survey was omitted. It should be noted that many of the questions in the survey were of an emotional nature and found to serve as triggers that could potentially contribute to psychological distress. After understanding this aspect of the survey in the pilot phase (described below), we set up a debriefing protocol after the survey was completed along with a province-wide counselling program if needed.

**Table 1:** Demographic characteristics of study participants.

Characteristics		Characteristics	
Age (years):	38 (32–43)	Education:	
18–25	27 (6%)	Less than high school	132 (31%)
26–40	268 (56%)	High school or higher	296 (69%)
>40	181 (38%)	Annual household income:	
Ethnic background:		<20K	188 (46%)
African	219 (47%)	20–40K	125 (31%)
Caribbean	55 (12%)	>40K	92 (23%)
European-British	57 (12%)	Years since HIV diagnosis:	7 (4–12)
French-Canadian	52 (11%)	HIV risk factors:	
Aboriginal	38 (8%)	Sex with men	321 (68%)
Other	48 (10%)	IDU	44 (9%)
Birth place:		Blood transfusion/blood product	36 (8%)
Africa	211 (44%)	Vertical transmission	4 (1%)
Canada	186 (39%)	Other	50 (11%)
Caribbean	49 (10%)	Unknown	62 (13%)
Other	31 (6%)	Hepatitis C	69 (15%)
Years in Canada:	14 (3–33)	Hepatitis B	16 (3%)
Region in Ontario:		Recent CD4 count:	466 (320–690)
Toronto	254 (52%)	≥200 cells/mm^3^:	317 (90%)
Ottawa	66 (13%)	Recent VL (log_10_ copies/mL):	3.8 (2.9–4.5)
Central West	58 (12%)	Ever on HIV medication:	413 (86%)
Central East	9 (2%)	When started:	Apr 04 (Jan 00-Sep 06)
Southwest	66 (13%)	Currently on HIV treatment:	356 (74%)
Northern	19 (4%)	Years on treatment:	4.5 (1.9–9.2)
Eastern	18 (4%)	Partner:	
Religion:		No partner	159 (33%)
Christian	147 (31%)	HIV negative	164 (34%)
Catholic	127 (27%)	HIV positive	114 (24%)
Protestant	76 (16%)	Unknown	46 (10%)
Atheist/none	23 (5%)	Current relationship:	
Muslim	21 (4%)	In sexual relationship	264 (55%)
Aboriginal Traditional	17 (4%)	Monogamous relationship	249 (51%)
Other	65 (14%)	Current contraceptive use	174 (36%)
Sexual orientation:		Last pregnancy planned	176 (43%)
Heterosexual	407 (89%)	Fertility history:	
Lesbian/Bisexual	42 (9%)	Lifetime Pregnancies:	
Other	8 (2%)	0	58 (12%)
Work:		1	80 (18%)
Working	186 (39%)	2	95 (20%)
On government assistance	239 (50%)	≥3	235 (50%)
Marital Status:		Lifetime Births:	
Never married	135 (29%)	0	124 (26%)
Married or common-law partner	173 (38%)	1	127 (27%)
Divorced/widowed	115 (25%)	2	96 (20%)
Living with a partner (neither married nor common-law)	35 (8%)	≥3	131 (27%)

Continuous variables presented as medians with interquartile range; categorical variables presented as N (%). IDU, injection drug use; VL, viral load.

### Survey Instrument and Validation

A 189-item survey instrument, “The HIV Pregnancy Planning Questionnaire”, was created using the methods of Fowler for instrument development [36] and based on the framework for modeling fertility motivation developed by Miller and colleagues [37]. The framework is partially based on the Traits-Desires-Intentions-Behaviour (TDIB) sequence that is required for pregnancy [37]. Questions from four previously validated surveys were included in the instrument including the “Contraceptive Decisions of HIV Positive Women” survey by Ogilvie and colleagues [11], the “HIV Cost and Services Utilization Study” survey by Chen and colleagues [26], the “Center for Special Studies” survey in Sagamu, Nigeria by Oladapo and colleagues [18] and the “Pregnancy Planning Instrument used for non-pregnant women” by Morin and colleagues [38]. The survey consisted of 12 domains including: 1) interest/desire to have children, 2) intent to have children in the future, 3) behaviour related to the pursuit of fertility, 4) menstrual, birth control and sexual history, 5) pregnancy and birth history, 6) perceived support for becoming pregnant, 7) satisfaction with providers, 8) needs assessment, 9) HIV medical history, 10) demographics, 11) anxiety and depression and 12) HIV stigma (full survey instrument available upon request).

The survey was first developed in English, and then translated into French using the back translation method [39]. Content validity was achieved by using items from the four previously validated surveys mentioned above [11], [18], [26], [38], by developing items based on an extensive literature search exploring factors that determined reproductive decisions in HIV-positive women [5]-[33], and by reviewing the questions with a project advisory panel of experts including HIV specialists, obstetricians, midwives and community members. Face validity was achieved by initially piloting the survey with 20 HIV-positive women in two focus groups. The focus group participants confirmed that the survey instrument items actually appeared to be measuring the intended items related to the domains. Furthermore, participants were asked to comment on each item in terms of comprehension, clarity and relevance. Further pilot testing was carried out with an initial 52 HIV-positive women that met the inclusion criteria and factor analysis was carried out to further determine the psychometric properties of the survey allowing adjustments prior to full implementation. Internal consistency of response was assessed by including an identical question twice in the survey using Cohen's kappa [11], [40].

### Statistical Analysis

Survey data was entered twice and verified prior to analysis. Statistical analyses were performed using SAS Version 9.1.3 (SAS Institute, Cary, North Carolina, USA). Baseline characteristics of the study population were summarized using medians and interquartile ranges (IQR) for continuous variables and frequencies and proportions for categorical variables. The geographic distribution of the study population was compared to the distribution of HIV-positive women living in Ontario using the chi-square test.

The primary outcome of interest was intention of pregnancy. Secondary outcomes included desire and actions taken to become pregnant. The question used to represent pregnancy intention, based on previous surveys [11], [18], [26], [38], was “How many children do you expect to give birth to in the future?” The variable was dichotomized into “no intention” if answered “0” and “intends pregnancy” if answered “1 or greater”. If the woman did not answer this question but responded “Never” to the question regarding when in the future she planned to be pregnant, she was assumed to have no intention of pregnancy. Women who did not answer that question but provided a time frame for future pregnancies were assumed to have intention of pregnancy in the future. The question used to represent pregnancy desire, based on previous surveys [11], [18], [26], [38], was “How many children would you like to give birth to in the future?” The variable was dichotomized into “no desire” if answered “0” and “desires pregnancy” if answered “1 or greater”. Similarly, missing data for women who did not answer that question was filled in using data from the question asking about timing of future desired pregnancies. Additional outcomes assessed included timing and number of expected pregnancies. These outcomes have been reported using medians and IQR for continuous variables and proportions for categorical variables.

Univariate logistic regression models were fit to determine the unadjusted odds ratios with 95% CIs for predictors of the primary outcome, pregnancy intention. The final multivariable logistic regression models included covariates that were a priori believed to be related to future intentions to have a child, such as age and number of live births. Additional variables which were significant at p<0.20 in the univariate analyses were candidates for inclusion in final multivariable logistic regression models for pregnancy intention. When multiple covariates measured similar phenomenon (e.g. ethnic background and country of birth), the variable representing each construct with the most statistical significance was chosen. Finally, the qualifying variables were entered into the multivariable model and finally selected by stepwise-selection method. Only the predictors with a significant effect (p<0.05) remained in the final multivariable model.

## Results

### Participants and Survey Validation

A total of 493 HIV-positive women living in Ontario, Canada were recruited from the 38 sites in Ontario. Three participants did not meet the inclusion criteria (one was over the age of 52, and two were not living in Ontario). Therefore, 490 surveys were included in the final analysis. The mirror question had 99.4% of observations in agreement (487/490) with a Cohen's kappa of 0.88 [95% CI 0.74–1.00] indicating excellent agreement between responses [11], [40]. The factor analyses of the pilot survey did not result in any modifications.

The final study population of 490 HIV-positive women living in Ontario, Canada had a median age of 38 (IQR, 32–43, range, 18–52). Of these, 61% were born outside of Canada, 52% were living in Toronto, 47% defined themselves as being of African ethnicity and 74% were currently on ART. Twenty-nine percent indicated that they had never been married and 51% stated they were in a monogamous relationship. Eighty-eight percent had previously experienced at least one pregnancy and 74% had given birth (31% after testing HIV positive). Only 15% of our population was co-infected with hepatitis C, consistent with Ontario statistics [35]. The comparison of the geographic distribution of the study population to that of the current distribution of HIV-positive women living in Ontario is presented in Figure 1 [35]. Furthermore, the recruitment matched the ethnicity distribution of the province [41]. Table 1 further summarizes the demographic characteristics of the entire cohort.

### Fertility Desires, Intentions and Actions

Using the TDIB model for fertility motivation, we have presented the participants' desires to become pregnant followed by their intentions and actions in Table 2. Of 490 women surveyed, 25 (5%) did not answer the question regarding fertility desire but fertility desire was determined for 10 of these women from the question regarding the desiring timing of future pregnancies. Of the resulting 475 women with fertility desire responses, 69% (95% CI, 64%–73%) stated positively that they would like to give birth in the future. Thirty-three women did not answer the question regarding fertility intentions, but intentions were determined from the question regarding the intended timing of future pregnancies for eight women. Of the resulting 465 women with fertility intention responses, 58% (95% CI, 53%–62%) stated positively that they were expecting to give birth in the future. Approximately half of those desiring and intending to have children in the future had taken action to become pregnant (Table 2). In total, 32% had approached their partner, 26% had spoken to their doctor about pregnancy and 12% had stopped birth control. The median number of children expected in the future was 1 (IQR, 0–2). Twenty percent of women expected the pregnancies to be within one year, 12% between one and two years and 7% between two and four years. Of the 315 women who desired to give birth and who had data on their intentions, 54 did not intend to give birth in the future (Table S1). These women tended to be older (>40), of British, European or French-Canadian ethnicity and hepatitis C co-infected (Table S2).

**Table 2:** Desires, intentions and actions of HIV-positive women to become pregnant.

Characteristics	N (%)
Fertility Desires	
Number of children desired in future:	
0	142 (31%)
1	147 (32%)
2	122 (26%)
>2	54 (12%)
Fertility Intentions	
Number of children intended in future:	
0	194 (42%)
1	121 (26%)
2	101 (22%)
>2	41 (9%)
Fertility Actions	
About having a baby:	
I have approached my partner	146 (32%)
My partner has approached me	138 (30%)
I have spoken to my doctor	123 (26%)
I have stopped using a birth control method in the past 12 months for getting pregnant	54 (12%)
General behavior in regard to a possible pregnancy:	
Try to avoid getting pregnant	225 (53%)
Don't do anything	150 (35%)
Try to get pregnant	39 (9%)
Currently pregnant	13 (3%)
Current behavior in regard to a possible pregnancy:	
Birth control every time I have sex to avoid getting pregnant	185 (49%)
Not use birth control and wouldn't be unhappy about getting pregnant	26 (7%)
Not use birth control but I am not trying to get pregnant	101 (27%)
Not use birth control and I am trying to get pregnant	31 (8%)

### Uni- and Multi-Variable Analysis for Fertility Intention

In the univariable analysis, HIV-positive women who intend to have children were more likely to be younger, of African ethnicity, not born in Canada, in Canada for a shorter duration, living in Toronto, diagnosed with HIV for a shorter duration, taking ART for a shorter duration, never married and were less likely to have acquired HIV through needle sharing, be co-infected with hepatitis B and C and have previously been pregnant and have given birth (Table 3). Covariates with no significant impact on fertility intentions included religion, employment status, education level, income, contraceptive use, previous planned pregnancy, marital status and sero-status of partner. Dividing African ethnicity into individual African countries did not influence the results (Table S3).

**Table 3:** Distribution of study participants by intention to have children in the future.

Characteristics	Intend Children		Unadjusted		Adjusted	
	Yes (N = 266)	No (N = 199)	Odds Ratio (95% CIs)	p-value	Odds Ratio (95% CIs)	p-value
Age						
Age: mean (±SD)	34.6±6.4	41.1±7.4	0.87 (0.84, 0.90)	<0.0001		
Age (piecewise)^a^						
18–25					1.08 (0.80, 1.45)	0.62
25–40					0.95 (0.89, 1.02)	0.15
>40					0.71 (0.63, 0.80)	<0.0001
Ethnic background:						
African	162 (78%)	45 (22%)	1		1	
Caribbean	33 (65%)	18 (35%)	0.51 (0.26, 0.99)	0.05	0.34 (0.15, 0.73)	0.006
European-British/French-Canadian	25 (25%)	77 (75%)	0.09 (0.05, 0.16)	<0.0001	0.10 (0.05, 0.20)	<0.0001
Aboriginal/Other	40 (48%)	44 (52%)	0.25 (0.15, 0.43)	<0.0001	0.27 (0.13, 0.54)	0.0002
Birth place:						
Africa	154 (77%)	45 (23%)	1			
Canada	61 (34%)	117 (66%)	0.15 (0.10, 0.24)	<0.0001		
Caribbean	30 (67%)	15 (33%)	0.58 (0.29, 1.18)	0.13		
Other	12 (40%)	18 (60%)	0.19 (0.09, 0.43)	<0.0001		
Years in Canada (for those not born in Canada): Median (IQR)	7 (2–20)	33 (12.8–42.8)	0.94 (0.92, 0.95)	<0.0001		
Region in Ontario:						
Toronto	179 (74%)	64 (26%)	1		1	
Non-Toronto	87 (39%)	135 (61%)	0.25 (0.17, 0.37)	<0.0001	0.40 (0.24, 0.68)	0.0007
Marital status:						
Never married	83 (66%)	42 (34%)	1			
Married or common-law Partner	99 (60%)	67 (40%)	0.75 (0.46, 1.21)	0.24		
Divorced/widowed	54 (49%)	57 (51%)	0.48 (0.28, 0.81)	<0.01		
Living with a partner (neither married nor common-law)	15 (44%)	19 (56%)	0.40 (0.18, 0.86)	0.02		
Annual household income in CAD:						
<20K	95 (54%)	82 (46%)	1.24 (0.75, 2.05)	0.41		
20–40K	73 (62%)	45 (38%)	1.73 (1.00, 3.02)	0.05		
>40K	44 (48%)	47 (52%)	1			
Years since HIV diagnosis: Median (IQR)	6 (3–10)	9 (5–15)	0.90 (0.87, 0.94)	<0.0001		
IDU:	11 (26%)	31 (74%)	0.23 (0.11, 0.47)	<0.0001		
Hepatitis C co-infected	17 (27%)	47 (73%)	0.23 (0.13, 0.42)	<0.0001		
Recent CD4 Count ≥200 (cells/mm^3^):	174 (58%)	126 (42%)	1.38 (0.68, 2.81)	0.37		
Years on HIV medication: Median (IQR)	3.9 (1.5–7.4)	6.1 (2.4–10.8)	0.92 (0.88, 0.96)	<0.001		
Current Relationship						
In sexual relationship	151 (59%)	104 (41%)	1.24 ( 0.86, 1.80)	0.26		
Monogamous relationship	133 (55%)	109 (45%)	0.84 (0.58, 1.21)	0.34		
Current contraceptive use	97 (58%)	71 (42%)	1.08 ( 0.74, 1.59)	0.68		
Last pregnancy planned	93 (56%)	73 (44%)	1.00 ( 0.67, 1.50)	0.99		
Fertility History						
Lifetime pregnancies:						
0–1	87 (64%)	48 (36%)	1.53 (1.01, 2.31)	0.05		
≥2	171 (54%)	144 (46%)	1			
Lifetime births:						
0–1	151 (63%)	89 (37%)	1.64 (1.13, 2.37)	<0.01	1.87 (1.11, 3.16)	0.02
≥2	111 (51%)	107 (49%)	1		1	

a Categorized age as a continuous variable in the model; SD, standard deviation; CIs, confidence intervals; IQR, interquartile range; IDU, injection drug user. Row percents presented for univariate analysis.

**Figure 2:**
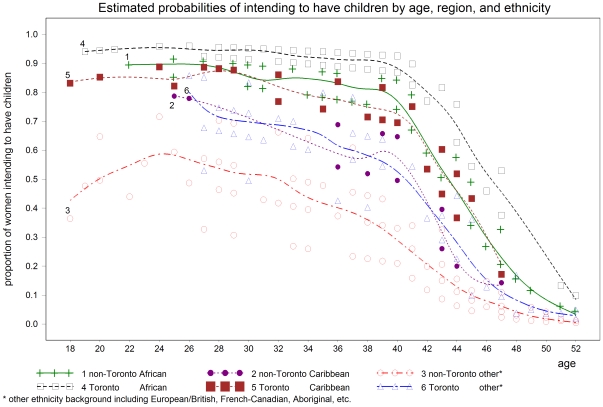
Predicted probability of intention to become pregnant based on age, Toronto residence or not, and ethnic background from the piecewise multivariable logistic regression model.

The results from the multivariable logistic regression modeling revealed that independent predictors of intendions to have children for HIV-positive women of reproductive age living in Ontario, Canada were age, ethnic background, living in Toronto and number of lifetime births (Table 3). Therefore, an HIV-positive woman in her 20 s or 30 s, of African descent, living in Toronto, who had already given birth to no more than one child, would be most likely to intend to become pregnant (Figure 2).

## Discussion

In this cross-sectional study of 490 HIV-positive women of reproductive age living in Ontario, Canada, we found that 69% desire and 58% intend to become pregnant in the future. These proportions are higher than the previous Canadian and American reports with similar inclusion criteria of fertility intentions of 26% and 29%, respectively [11], [26]. The differences between our results and the previous Canadian study [11] conducted in the province of British Columbia likely relate to the unique epidemiology of HIV infection in that province, where the population includes a significant number of drug users (64%), high rates of hepatitis C co-infection (65%) and a high proportion of women who identify as Aboriginal (43%); all variables which were associated with lower fertility intentions in our study. The previous American study [26] was carried out in a population enrolled in 1996 and with a second follow-up in 1997 and 1998, a period prior to the wide use and successes of ART, likely accounting for much of the discrepancy. Furthermore, the demographic characteristics of the HIV-positive women in the United States differ from Canada with fewer immigrants and more African Americans and Latinas being affected than in Canada. Our proportions are very similar to reports of fertility desires and intentions reported in African studies. In a Nigerian study, the researchers found that 68% and 65.5% of HIV-positive women desired and intended to have children [18]. This could relate to the fact that many HIV-positive women living in Ontario, Canada were born in Africa and have sought refugee status in or immigrated to Canada (47% identified as African ethnicity). Finally, in a study of the general Canadian female population, 37.5% reported intention of pregnancy [42]. The higher proportions in our study may reflect our focus on women of reproductive age and ethnicity differences between the two populations.

Predictors of intentions to become pregnant in our study included age, ethnic background, geographic region and pregnancy history. Thus, women less than age 40, of African ethnicity, who lived in Toronto and who have given birth to no more than one child were most likely to intend to become pregnant. Younger age has consistently been a predictor of fertility intentions in all studies of HIV-positive women [5]–[33]. This is of clinical importance as many new HIV infections in Ontario are occurring in younger women [35]. In our study, women of African ethnicity more likely intended to become pregnant, possibly because of the cultural importance of parenting to this ethnic group [43]–[45]. While other studies have demonstrated the important differences in fertility rates between different African countries [46], our study did not find such differences in fertility intentions; however, this conclusion is limited by our sample size. A newly identified predictor of fertility intentions in our study was the current geographic region of residency. More women living in the large urban area of Toronto intended to become pregnant and the reason is unclear; this could possibly be related to differences in demographic characteristics or access to care and services. These will be important issues to flesh out for the implementation of healthcare programs and policies on fertility for HIV-positive individuals.

There are a number of important implications for our results on healthcare services that providers and policy makers should consider. In large urban cities with high rates of immigration from Africa, such as Toronto, a significant number of HIV-positive women will intend to become pregnant. The intention of these women to become pregnant was not influenced by their marital status or by their religion, employment status, education level, income or clinical HIV status. Healthcare providers need to consider preparing to support these women to plan for safer pregnancies in order to maximize the health of the women, and to protect the future children by reducing vertical transmission. Prior work in the area of HIV and fertility has focused on pregnancy and not on the pre-conception pregnancy planning stage. It is important for clinicians to recognize that many HIV-positive women desire and intend to become pregnant and to discuss pregnancy plans with their patients and partners prior to conception. Clinicians should be prepared to provide information on general pre-conception guidelines, methods to increase pregnancy success and decrease horizontal HIV transmission as well as general information on prenatal care and the appropriate use of ART during pregnancy. Guidelines and counselling tools would be useful to provide this accurate non-judgmental reproductive health information in the pre-conception period. Such guidelines and tools are being developed in Canada [47]. Our findings may also provide insight for other developed countries that have a significant number of individuals who seek refugee status and immigration from Africa including England, France, Germany, Spain, and Australia.

The present study has a number of limitations including the risk of some degree of selection bias due to the high literacy level required for the survey, which was limited to two languages, English and French; for many women those languages are not their mother tongue. Women who desire and intend to give birth in the future may be more likely to complete a questionnaire on this topic. The women were in general healthy, with high CD4 counts and with viral suppression on ART. The study was self-administered allowing women the privacy to complete questions on very private subjects. While this likely contributes to women answering more honestly, it also likely led to some unanswered questions.

There were also a number of strengths in our study including its large sample size. An attempt was made to match the study recruitment to the geographic distribution of HIV-positive women living in Ontario, allowing for generalizability to the Ontario female population and allowing for more meaningful contributions to provincial policy. A significant proportion of the enrolment was carried out using a community-based research model involving ASOs and women living with HIV in the study recruitment and coordination. This had multiple benefits including engaging participants who do not usually partake in research and increasing the capacity of the Ontario HIV community to conduct research.

The results of this study are the first step to developing a program on fertility and pregnancy planning for HIV-positive individuals in Ontario and Canada [47], [48]. Additional planned projects include a review of the services provided by Canadian fertility clinics for HIV-positive individuals, and an exploration of Canadian healthcare providers' attitudes towards fertility and pregnancy for HIV-positive individuals [48]. The development of national guidelines on pregnancy planning as well as provincial and national HIV Fertility Programs, including a knowledge translation plan, are underway [47]. The aim of all these endeavours is to support individuals living with HIV in Ontario and Canada with their fertility and pregnancy planning needs in an ethical and holistic manner. We hope that our research and ongoing projects assist HIV-positive individuals, policy makers and healthcare providers globally to develop their programs for safer, supportive pregnancy planning for HIV-positive individuals in their communities. Although much research has been started on a global level in the area of safer pregnancy planning, fertility, pregnancy, and parenting [49]–[56], significantly more is needed to support HIV-positive individuals to have healthy pregnancies and families. Finally, similar research should be carried out with HIV-positive male populations, which is being planned by our research team.

## Supporting Information

Table S1 Demographic characteristics of study participants who desire birth but do not intend birth.
(0.11 MB DOC)

Table S2 Distribution of study participants who desired to have children by intention to have children in the future.
(0.11 MB DOC)

Table S3 Distribution of specific African countries where African participants were born.
(0.04 MB DOC)
