# Supplementary material for: Fertility Desires and Intentions of HIV-Positive Women of Reproductive Age in Ontario, Canada: A Cross-Sectional Study
Source: PLoS One. 2009 Dec 7;4(12):e7925. doi: 10.1371/journal.pone.0007925 (PMC2785467; doi:10.1371/journal.pone.0007925)
Supplement: Table S1 — Demographic characteristics of study participants who desire birth but do not intend birth. (0.11 MB DOC) [file pone.0007925.s001.doc]

**Table S1:** Demographic characteristics of study participants who desire birth but do not intend birth

| *Characteristics* | | *Total sample* | |
| --- | --- | --- | --- |
|  | | *N* | *Total (N=54)* |
| Age (years) | | 53 | 41 (33-45) |
|  | 18-25 |  | 2 (4%) |
|  | 26-40 |  | 24 (45%) |
|  | >40 |  | 27 (51%) |
| Ethnic background | |  |  |
|  | African |  | 8 (16%) |
|  | Caribbean |  | 4 (8%) |
|  | European-British |  | 12 (24%) |
|  | French-Canadian |  | 11 (22%) |
|  | Aboriginal |  | 8 (16%) |
|  | Other |  | 8 (16%) |
| Birth place | |  |  |
|  | Africa |  | 8 (15%) |
|  | Canada |  | 40 (75%) |
|  | Caribbean |  | 2 (4%) |
|  | Other |  | 3 (6%) |
| Years in Canada | | 53 | 33 (28-41) |
| Region in Ontario | |  |  |
|  | Central East |  | 1 (2%) |
|  | Central West |  | 5 (9%) |
|  | Eastern and other |  | 4 (7%) |
|  | Northern |  | 9 (17%) |
|  | Ottawa |  | 7 (13%) |
|  | Southwestern |  | 9 (17%) |
|  | Toronto |  | 19 (35%) |
| Religion | |  |  |
|  | Aboriginal Traditional |  | 5 (10%) |
|  | Atheist/none |  | 4 (8%) |
|  | Catholic |  | 20 (39%) |
|  | Christian |  | 10 (20%) |
|  | Muslim |  | 1 (2%) |
|  | Protestant |  | 6 (12%) |
|  | Other |  | 5 (10%) |
| Sexual orientation | |  |  |
|  | Heterosexual |  | 39 (75%) |
|  | Lesbian/Bisexual |  | 10 (19%) |
|  | Other |  | 3 (6%) |
| Working | |  | 18 (34%) |
| On government assistance | |  | 35 (66%) |
| Marital status | |  |  |
|  | Divorced/widowed |  | 11 (21%) |
|  | Living with a partner (neither married nor common-law) |  | 8 (15%) |
|  | Married or common-law partner |  | 17 (33%) |
|  | Never married |  | 16 (31%) |
| Education | |  |  |
|  | High school or higher |  | 31 (61%) |
|  | Less than high school |  | 20 (39%) |
| Annual household income | |  |  |
|  | 20-40K |  | 14 (30%) |
|  | <20K |  | 21 (45%) |
|  | >40K |  | 12 (26%) |
| Years since diagnosis of HIV positive | | 50 | 9 (4-14) |
| Risk Factor | |  |  |
|  | Sex with men |  | 38 (72%) |
|  | IDU |  | 12 (23%) |
|  | Blood Transfusion/blood product |  | 5 (9%) |
|  | Vertical transmission |  | 0 (0/53) |
|  | Other |  | 4 (8%) |
|  | Unknown |  | 5 (9%) |
| Hepatitis C | |  | 19 (36%) |
| Recent CD4 count | | 43 | 471 (340-700) |
|  | ≥200 cells/mm^3^ |  | 41 (95%) |
| Recent VL (log_10_ copies/mL) | | 14 | 3.5 (3.3-4.1) |
| Ever on HIV medication | |  | 45 (83%) |
|  | When started | 44 | Aug01 (Jun97-Apr07) |
| Currently on HIV treatment | |  | 40 (74%) |
| Years on treatment | | 43 | 6.5 (1.5-11.3) |
| Partner | |  |  |
|  | HIV negative |  | 25 (46%) |
|  | HIV positive |  | 8 (15%) |
|  | No Partner |  | 19 (35%) |
|  | Unknown |  | 2 (4%) |
| Current Relationships | |  |  |
|  | in sexual relationship |  | 25 (46%) |
|  | monogamous relationship |  | 28 (52%) |
| Current contraception use | |  | 15 (28%) |
| Last pregnancy planned | |  | 17 (37%) |
| Fertility history | |  |  |
| Lifetime pregnancies | |  |  |
|  | 0 |  | 6 (11%) |
|  | 1 |  | 7 (13%) |
|  | 2 |  | 7 (13%) |
|  | ≥3 |  | 33 (62%) |
| Lifetime births | |  |  |
|  | 0 |  | 15 (28%) |
|  | 1 |  | 12 (23%) |
|  | 2 |  | 10 (19%) |
|  | ≥3 |  | 16 (30%) |

VL, viral load.
