# Supplementary material for: Fertility Desires and Intentions of HIV-Positive Women of Reproductive Age in Ontario, Canada: A Cross-Sectional Study
Source: PLoS One. 2009 Dec 7;4(12):e7925. doi: 10.1371/journal.pone.0007925 (PMC2785467; doi:10.1371/journal.pone.0007925)
Supplement: Table S2 — Distribution of study participants who desired to have children by intention to have children in the future. (0.11 MB DOC) [file pone.0007925.s002.doc]

**Table S2:** Distribution of study participants who desired to have children by intention to have children in the future

| *Characteristics (who desired to have children) (N = 315)* | | *Intend Children* | |  | *Unadjusted* | |  | *Adjusted* | |
| --- | --- | --- | --- | --- | --- | --- | --- | --- | --- |
|  |  | *Yes (N = 261)* | *No N = 54)* |  | *Odds Ratio  (95% CIs)* | *p-value* |  | *Odds Ratio  (95% CIs)* | *p-value* |
| Age | |  |  |  |  |  |  |  |  |
|  | Mean ± SD | 34.5 ± 6.3 | 39.4 ± 8.1 |  | 0.89 (0.85, 0.94) | <0.0001 |  |  |  |
|  | ≤40 | 204 (89%) | 26 (11%) |  | 4.51 (2.41, 8.42) | <0.0001 |  | 6.53 (2.93, 14.56) | <0.0001 |
|  | >40 | 47 (64%) | 27 (36%) |  | 1 |  |  | 1 |  |
| Ethnic Background | |  |  |  |  |  |  |  |  |
|  | African | 158 (95%) | 8 (5%) |  | 18.93 (7.6, 47.11) | <0.0001 |  | 13.39 (4.75, 37.74) | <0.0001 |
|  | Caribbean | 33 (89%) | 4 (11%) |  | 7.91 (2.42, 25.86) | 0.0006 |  | 4.54 (1.20, 17.21) | 0.03 |
|  | European-British/French-Canadian | 24 (51%) | 23 (49%) |  | 1 |  |  | 1 |  |
|  | Aboriginal/Other | 40 (71%) | 16 (29%) |  | 2.4 (1.06, 5.41 ) | 0.04 |  | 1.82 (0.71, 4.66) | 0.21 |
| Birth Place | |  |  |  |  |  |  |  |  |
|  | Africa | 150 (95%) | 8 (5%) |  | 12.5 (5.53, 28.27) | <0.0001 |  |  |  |
|  | Canada | 60 (60%) | 40 (40%) |  | 1 |  |  |  |  |
|  | Caribbean | 30 (94%) | 2 (6%) |  | 10 (2.26, 44.2) | 0.002 |  |  |  |
|  | Other | 12 (30%) | 3 (20%) |  | 2.67 (0.71, 10.05) | 0.15 |  |  |  |
| Years in Canada (for those not born in Canada): Median (IQR) | | 7 (2-20) | 33 (28-41) |  | 0.91 (0.89, 0.93) | <0.0001 |  |  |  |
| Region in Ontario | |  |  |  |  |  |  |  |  |
|  | Toronto | 175 (90%) | 19 (10%) |  | 1 |  |  |  |  |
|  | Non-Toronto | 86 (71%) | 35 (29%) |  | 0.27 (0.14, 0.49) | <0.0001 |  |  |  |
| On government assistance | |  |  |  |  |  |  |  |  |
|  | Yes | 121 (78%) | 35 (22%) |  | 2.07 (1.11, 3.85) | 0.02 |  |  |  |
|  | No | 129 (88%) | 18 (12%) |  | 1 |  |  |  |  |
| Marital Status | |  |  |  |  |  |  |  |  |
|  | Never married | 80 (83%) | 16 (17%) |  | 2.67(0.97, 7.34) | 0.06 |  |  |  |
|  | Married or common-law Partner | 99 (85%) | 17 (15%) |  | 3.11 (1.14, 8.45) | 0.03 |  |  |  |
|  | Divorced/widowed | 52 (83%) | 11 (17%) |  | 2.52 (0.86, 7.4) | 0.09 |  |  |  |
|  | Living with a partner (neither married nor common-law) | 15 (65%) | 8 (35%) |  | 1 |  |  |  |  |
| Education | | |  |  |  |  |  |  |  |
|  | High school or higher | 165 (84%) | 31 (16%) |  | 1.69 (0.9, 3.18) | 0.1 |  |  |  |
|  | Less than high school | 63 (76%) | 20 (24%) |  | 1 |  |  |  |  |
| Injection drug use | |  |  |  |  |  |  |  |  |
|  | Yes | 10 (45%) | 12 (55%) |  | 0.14 (0.06, 0.35) | <0.0001 |  |  |  |
|  | No | 243 (86%) | 41 (14%) |  | 1 |  |  |  |  |
| Hepatitis C co-infected | |  |  |  |  |  |  |  |  |
|  | Yes | 15 (44%) | 19 (56%) |  | 0.12 ( 0.05, 0.25) | <.0001 |  | 0.26 (0.10, 0.66) | 0.005 |
|  | No | 231 (87%) | 34 (13%) |  | 1 |  |  | 1 |  |
| Years since diagnosis as HIV positive: Median (IQR) | | 6 (3-10) | 9 (5-15) |  | 0.92 ( 0.87, 0.97) | <.01 |  |  |  |
| Years on HIV medication: Median (IQR) | | 3.9 (1.5-7.4) | 6.5 (1.5-11.3) |  | 0.91 ( 0.85, 0.98) | <.01 |  |  |  |
| Fertility History | |  |  |  |  |  |  |  |  |
| Lifetime pregnancies | |  |  |  |  |  |  |  |  |
|  | 0-1 | 87 (87%) | 13 (13%) |  | 1.61 ( 0.82, 3.17) | 0.17 |  |  |  |
|  | ≥2 | 166 (81%) | 40 (19%) |  | 1 |  |  |  |  |
| Lifetime births | |  |  |  |  |  |  |  |  |
|  | 0-1 | 150 (85%) | 27 (15%) |  | 1.35 ( 0.75, 2.44) | 0.32 |  |  |  |
|  | ≥2 | 107 (80%) | 26 (20%) |  | 1 |  |  |  |  |

SD, standard deviation; CIs, confidence intervals; IQR, interquartile range.
