# Supplementary material for: Fertility Desires and Intentions of HIV-Positive Women of Reproductive Age in Ontario, Canada: A Cross-Sectional Study
Source: PLoS One. 2009 Dec 7;4(12):e7925. doi: 10.1371/journal.pone.0007925 (PMC2785467; doi:10.1371/journal.pone.0007925)
Supplement: Table S3 — Distribution of specific African countries where African participants were born. (0.04 MB DOC) [file pone.0007925.s003.doc]

**Table S3:** Distribution of specific African countries where African participants were born

| *African countries* | *Sample* | *Intend Children* | |
| --- | --- | --- | --- |
|  | *(N=194)* | *yes (N=150)* | *no (N=44)* |
| Burundi | 11 (6%) | 6 (55%) | 5 (45%) |
| Cameroon | 8 (4%) | 7 (88%) | 1 (12%) |
| Congo | 14 (7%) | 11 (79%) | 3 (21%) |
| Ethiopia | 20 (10%) | 16 (80%) | 4 (20%) |
| Ghana | 5 (3%) | 5 (100%) | 0 (0%) |
| Kenya | 9 (5%) | 7 (78%) | 2 (22%) |
| Nigeria | 5 (3%) | 5 (100%) | 0 (0%) |
| Rwanda | 12 (6%) | 9 (75%) | 3 (25%) |
| South Africa | 8 (4%) | 8 (100%) | 0 (0%) |
| Swaziland | 5 (3%) | 4 (80%) | 1 (20%) |
| Uganda | 17 (9%) | 14 (82%) | 3 (18%) |
| Zambia | 8 (4%) | 6 (75%) | 2 (25%) |
| Zimbabwe | 47 (24%) | 31 (66%) | 16 (34%) |
| Countries in which patients <5 | 25 (13%) | 21 (84%) | 4 (16%) |

Chi-square test p-value = 0.48; Only 5 women born in Africa had missing data for the specific country that they were born; Census Canada regulations do not allow reporting on any individual country where less than five individuals report they were born, and for confidentiality reasons we have applied the same standards to our reporting.
